# A conditional multi-trait sequence GWAS discovers pleiotropic candidate genes and variants for sheep wool, skin wrinkle and breech cover traits

**DOI:** 10.1186/s12711-021-00651-0

**Published:** 2021-07-08

**Authors:** Sunduimijid Bolormaa, Andrew A. Swan, Paul Stothard, Majid Khansefid, Nasir Moghaddar, Naomi Duijvesteijn, Julius H. J. van der Werf, Hans D. Daetwyler, Iona M. MacLeod

**Affiliations:** 1 Agriculture Victoria, AgriBio, Centre for AgriBioscience, Bundoora, VIC 3083 Australia; 2 Cooperative Research Centre for Sheep Industry Innovation, Armidale, NSW 2351 Australia; 3 Animal Genetics and Breeding Unit, University of New England, Armidale, NSW 2351 Australia; 4 Faculty of Agricultural, Life & Environmental Sciences, University of Alberta, Edmonton, AB T6G 2R3 Canada; 5 School of Environmental and Rural Science, University of New England, Armidale, NSW 2351 Australia; 6 Hendrix Genetics, Boxmeer, The Netherlands; 7 School of Applied Systems Biology, La Trobe University, Bundoora, VIC 3086 Australia

## Abstract

**Background:**

Imputation to whole-genome sequence is now possible in large sheep populations. It is therefore of interest to use this data in genome-wide association studies (GWAS) to investigate putative causal variants and genes that underpin economically important traits. Merino wool is globally sought after for luxury fabrics, but some key wool quality attributes are unfavourably correlated with the characteristic skin wrinkle of Merinos. In turn, skin wrinkle is strongly linked to susceptibility to “fly strike” (*Cutaneous myiasis*), which is a major welfare issue. Here, we use whole-genome sequence data in a multi-trait GWAS to identify pleiotropic putative causal variants and genes associated with changes in key wool traits and skin wrinkle.

**Results:**

A stepwise conditional multi-trait GWAS (CM-GWAS) identified putative causal variants and related genes from 178 independent quantitative trait loci (QTL) of 16 wool and skin wrinkle traits, measured on up to 7218 Merino sheep with 31 million imputed whole-genome sequence (WGS) genotypes. Novel candidate gene findings included the *MAT1A* gene that encodes an enzyme involved in the sulphur metabolism pathway critical to production of wool proteins, and the *ESRP1* gene. We also discovered a significant wrinkle variant upstream of the *HAS2* gene, which in dogs is associated with the exaggerated skin folds in the Shar-Pei breed.

**Conclusions:**

The wool and skin wrinkle traits studied here appear to be highly polygenic with many putative candidate variants showing considerable pleiotropy. Our CM-GWAS identified many highly plausible candidate genes for wool traits as well as breech wrinkle and breech area wool cover.

**Supplementary Information:**

The online version contains supplementary material available at 10.1186/s12711-021-00651-0.

## Background

More than half of the 70 million sheep in Australia are pure Merino that are traditionally bred for high value wool which is globally sought after for luxury fabric manufacture. The gross value of wool produced in Australia was 3 billion AU dollars in 2020 (https://woolproducers.com.au/about-us/wool-trade/). The value of a sheep’s wool fleece depends on several key attributes, including fleece weight, fibre diameter, staple strength and length, as well as crimp (or curvature) [1]. Each of these attributes are moderately to highly heritable and show some degree of genetic correlation between them. Some traits are correlated in a favourable direction (e.g. clean fleece weight and staple length [2]) while for other trait pairs there are significant unfavourable correlations that affect both wool quality (e.g. fleece weight and fibre diameter) and welfare traits (e.g. breech skin wrinkle and fibre diameter) [3]. While breech skin wrinkle and breech area wool cover are not of direct economic value, they are positively genetically correlated to susceptibility to “flystrike” (*Cutaneous myiasis*) [4]. When the skin around the sheep’s rear (breech area) is heavily wrinkled and woolly, this area is more likely to become damp and soiled by faeces (thus attracting flies to lay eggs) compared to less wrinkled and bare skin. When the eggs hatch the larvae can break the skin to feed on exudate (*Cutaneous myiasis*). Flystrike imposes a heavy economic and welfare burden in Australian sheep flocks and was recently estimated to cost the Australian industry over $170 million annually [5].

To our knowledge, only two genome-wide association studies (GWAS) have previously mapped quantitative trait loci (QTL) for a range of wool traits or skin wrinkle in sheep, however, these studies used only 50k [6] or high-density (600k) SNP array genotypes [7]. The estimated effect of a SNP on phenotype depends on the linkage disequilibrium (LD) between the SNP and the causal variant. Standard SNP arrays are very unlikely to include causal variants and, furthermore, the SNPs on arrays are preselected to be (highly) polymorphic across breeds. This can result in rarer or breed-specific causal variants that are not in strong LD with the array SNPs, and thus their effects may not be captured in GWAS. In contrast to SNP arrays, whole-genome sequence (WGS) data should include all or at least many of the causal variants. Recently, we demonstrated that the use of imputed whole-genome sequence (WGS) variants increased the accuracy of genomic selection for wool traits in sheep [8]. Thus, it is of considerable interest to undertake genome-wide association studies (GWAS) with imputed sequence to fine-map putative causal variants and genes underpinning economically important wool traits as well as breech wrinkle. This would provide a better understanding of the underlying biology and pleiotropy across traits. Also, variants with large effects for single traits or with multiple pleiotropic effects are of particular interest to enrich custom SNP panels to improve accuracy of genomic prediction [9].

Use of multi-trait meta-GWAS analysis that combines results from individual trait GWAS has been demonstrated to be beneficial for identifying pleiotropic variant effects among traits for SNP array genotypes [7, 10–12], but has not yet been tested for sequence-level GWAS in sheep. Pausch et al. [13] demonstrated that, while it is possible to identify causal variants using GWAS with accurately imputed WGS, precise mapping can be difficult due to long-range LD resulting in many variants being associated with each real causal variant. An alternative approach to GWAS for fine-mapping QTL is to fit all the variants simultaneously in a model, such as BayesR [14, 15] that accounts for linked markers, fits a sparse distribution of QTL effects and allows for diverse genetic architectures. With many thousands of individuals, BayesR with WGS becomes computationally challenging, however a subset of the sequence variants can be tested instead.

Therefore, the main objective of this study was to fine-map putative pleiotropic causal variants for 16 wool and breech wrinkle traits measured in 7218 Merino sheep. We did this by extending the approach of Bolormaa et al. [10] and undertook a stepwise conditional multi-trait GWAS (CM-GWAS) with 31 million WGS variants. We present a list of annotated putative causal variants and genes and indicate their pleiotropic effects across traits.

## Methods

### Phenotype data and traits

These Merino animals were sourced from the mixed breed Information Nucleus (IN) flock of the Cooperative Research Centre for Sheep Industry Innovation (Sheep CRC) [16, 17]. The project made available a “Q matrix” with breed proportion for each animal from among a range of breeds, as well as strains of Merino sheep, based on pedigree recording information [18]. For this study, we used only “pure” Merino (MER) animals, where “pure” was defined as the sum of their breed proportions being more than 90% Merino. Merinos were identified as belonging to one of three strains based on wool type: ultra-fine, fine to medium, and broad fibre diameter wool [18]. Up to 7218 animals were measured for 16 traits. Wool production traits were measured at two ages defined as “yearling” (150 days < Age < 550 days) and “adult” (Age ≥ 550 days) and these were considered as different traits. Subjective scores for breech wool cover and breech skin wrinkle were also analysed because they are important flystrike susceptibility indicator traits [4]. Not all sheep were measured for all traits. Trait definitions and numbers of records for each trait of the corrected phenotypes for the animals with phenotypes and genotypes are in Table 1. Phenotypes were obtained from the official Sheep Genetics industry genetic evaluation database [19], and were pre-adjusted for various fixed effects (contemporary group, flock, drop year, sex, birth type, and rearing type, age of measurement and age of dam) at AGBU, NSW, Australia. A complete description of the measurement and recording of wool production and quality assessments is in Hatcher et al. [20] and Bolormaa et al. [7].

**Table 1:** Trait names and acronyms (a = adult and y = yearling), number of records and units of measure

Trait acronym	Full description	Units of measure	Number of records
ygfw	Yearling greasy fleece weight	kg	6741
agfw	Adult greasy fleece weight	kg	4307
ycfw	Yearling clean fleece weight	kg	6105
acfw	Adult clean fleece weight	kg	3096
ysl	Yearling staple length	mm	4644
asl	Adult staple length	mm	3159
yfd	Yearling mean fibre diameter	μm	6359
afd	Adult mean fibre diameter	μm	3157
ydcv	Yearling fibre diameter coefficient of variation	%	6365
adcv	Adult fibre diameter coefficient of variation	%	3158
ycuv	Yearling mean fibre curvature	°/mm	4203
acuv	Adult mean fibre curvature	°/mm	3187
yss	Yearling staple strength	N/k tex	4639
ass	a. staple strength	N/k tex	3155
ebwr	Breech wrinkle	1–5 score	7218
ebcov	Breech cover	1–5 score	5747

### Whole-genome sequence genotype data

WGS genotypes of 7218 Merinos used in this study were imputed together with a larger group of sheep (47,000 animals), following Bolormaa et al. [21] and, which we briefly described here for clarity. The 47k animals were a mix of purebreds, crosses and composites were imputed together as part of a research project that demonstrated that the use of sequence variants increased the accuracy of genomic prediction [8]. The Merinos used in our study were jointly phased and imputed to whole-genome sequence because empirical testing showed that this approach surpassed the accuracy of imputing breed groups separately [21]. The reference sequence data had an average read depth of ~ 10×, and details of the bioinformatic pipeline for quality control and variant calling are described in Bolormaa et al. [21].

The Merino animals were previously genotyped using low-density (“12k”), medium-density (“50k”), or high-density (“HD” with ~ 500k) SNP panels. Quality control of SNP array genotypes, imputation of sporadic missing genotypes within each SNP panel, and imputation of the genotypes from lower density to medium density, and then to HD SNP panels are described in [11, 22]. The imputation from HD SNPs to WGS variants was performed using the Minimac3 algorithm (version 2.0.1; [23]). Minimac3 requires pre-phased genotypes, in both reference (WGS) and target sets. The Eagle (version 2.3; [24]) software was used for pre-phasing in both reference WGS and HD target sets. The WGS data of 726 animals representing multiple European breeds and crosses [25] were used as a reference set as detailed in Bolormaa et al. [21]. Prior to imputation, we removed variants from the reference sequence data with less than 5 minor allele counts, variants with more than 2 alleles (2.97 million), and the variants on the X chromosome, leaving 39,844,235 variants. After imputation to WGS, all imputed SNPs and indels with the Minimac3 *R*^2^ statistic higher than 0.4 were retained (removing approximately 20% of the variants), resulting in a final set of 31,154,082 imputed variants for the 47,000 sheep animals. Bolormaa et al. [21] showed that the Minimac3 *R*^2^ statistic is a good proxy for empirical imputation accuracy. According to their study, a Minimac3 *R*^2^ higher than 0.4 corresponded to the empirical imputation accuracy of 0.87 (measured as the correlation between real and imputed genotypes). This threshold does remove a large proportion of very rare variants because these are the most difficult to impute accurately. However, very rare variants can also result in false positive results in GWAS, thus we also imposed a MAF threshold for the GWAS. All variants were fully annotated for a range of characteristics using the NGS-SNP pipeline [26].

### Sequence GWAS

#### Single-trait GWAS

The mixed model used for the GWAS fitted each sequence variant as a covariate, one at a time, and tested for association with the trait:

1 $${\mathbf{y}} = {\mathbf{1}}_{\text{n}} \upmu + {\mathbf{s}}_{i} \upalpha _{i} + {\mathbf{Qq}} + {\mathbf{g}} + {\mathbf{e}},$$

where **y** is the vector of phenotypic values pre-adjusted for fixed effects of the animals, **1**_n_ is an ${\text{n}} \times 1$ vector of 1s (n = number of animals with phenotypes), μ is the overall mean, **s**_*i*_ is a vector of genotypes (coded as 0, 1, and 2) for each animal at the *i*-th variant, $\upalpha _{i}$ is the corresponding variant effect, **Q** is a matrix with Merino strain proportions calculated from the pedigree (${\mathbf{q}}\sim N(\mathbf{0},\;{\mathbf{I}}\upsigma _{{\text{q}}}^{2}$) [18], **g** is a vector of genomic breeding values (GEBV) $\sim N(\mathbf{0},\;{\mathbf{G}}\upsigma _{{\text{g}}}^{2}$), where $\upsigma _{{\text{g}}}^{2}$ is the genetic variance and **G** is the genomic relationship matrix (GRM) calculated from HD genotypes, and **q** and **e** are the vectors of random effects of Merino strain proportions and residual error, respectively. Merino strains included ultra-fine, fine to medium, and broad fibre diameter wools. For a variant to be included in the GRM, its minor allele frequency (MAF) had to be higher than 0.005 in the entire dataset. The analysis was performed using the Wombat software [27]. Variance components for random effects were first computed without fitting SNP effects (**s**_*i*_α_*i*_) in the model (Eq. 1) using Wombat. Following Bolormaa et al. [28], the false discovery rate (FDR) for variants associated with each trait at *P*-values of 10^–5^ and 10^–6^ was calculated as: ${\text{FDR}} = P\left( {1 - A/T} \right)/\left( {\left( {A/T} \right)\left( {1 - P} \right)} \right)$, where *P* is the *P*-value tested, *A* is the number of SNPs that were significant at the *P*-value tested and *T* is the total number of SNPs tested.

#### CM-GWAS

Here, we extend the multi-trait meta-analysis method (M-GWAS) described in Bolormaa et al. [10] to a conditional multi-trait meta-analysis (CM-GWAS). This analysis used WGS variant effects estimated from the 16 single-trait GWAS to identify pleiotropic variants that affected wool traits, breech wrinkle and breech cover. The multi-trait meta-analysis (M-GWAS) $\chi ^{2}$ statistic has degrees of freedom equal to the number of traits analysed and was calculated as described in [10]:

2 $${\text{multi-trait}} \chi ^{2} = {\mathbf{t^{\prime}}}_{i} {\mathbf{V}}^{{ - 1}} {\mathbf{t}}_{i} ,$$

where **t**_*i*_ is a vector of the signed t-values of the effects of the *i*-th SNP for the 16 traits and **V**^−1^ is the inverse of the 16 × 16 correlation matrix where the correlation was calculated over the all estimated SNP effects (signed t-values) between each pair of traits. Details of the multi-trait statistic property are in [10].

**Fig. 1:**
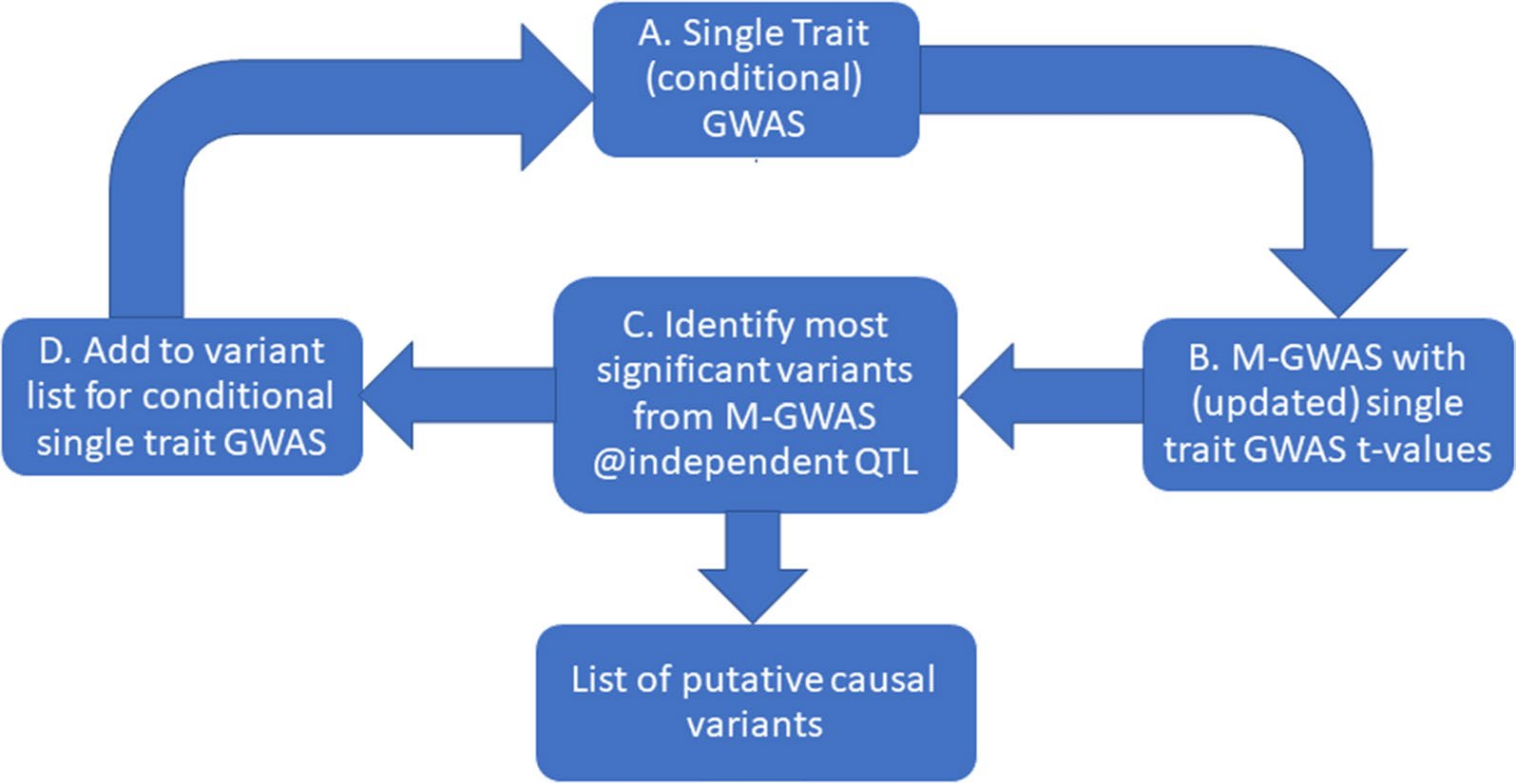
Workflow for the conditional multi-trait meta-GWAS (CM-GWAS). The CM-GWAS cycles between single-trait GWAS for all traits and a multi-trait meta-GWAS (M-GWAS). After the first cycle, steps from A to D are repeated, jointly fitting the most significant independent M-GWAS variants in a conditional single-trait GWAS for all traits. The t-values from the conditional single trait GWAS (step A) are then updated to re-run the M-GWAS (step B). This continues for one or more cycles until no more significant variants from the M-GWAS are detected in step C. At step C, to determine independence, first the most significant M-GWAS variant from each chromosome is selected and added to the list of putative causal variants. If the pairwise linkage disequilibrium (LD) between this variant and any other significant variant on the same chromosome has r^2^ > 0.1, these other variants are considered as potentially tagging the same causal variant and are not considered as independent QTL for this cycle. Then, from the remaining significant variants in LD r^2^ ≤ 0.1, the next most significant is selected on each chromosome, LD is tested between these and the remaining significant variants and so on, until no more significant variants can be identified in this cycle

The CM-GWAS approach cycles back and forward between the single-trait GWAS and M-GWAS to re-test variants conditional on jointly fitting the most significant putative causal variants from independent QTL regions (where we defined significant as *P* < 10^–5^). The independence of QTL regions was defined by LD levels among variants. The steps for the CM-GWAS are outlined below and visualized in Fig. 1.

1. A single-trait GWAS was performed for all 16 traits.
2. The signed t-values (**t**_*i*_ = each SNP effect from GWAS divided by its standard error) and correlation between 16 traits (**V** matrix) were calculated to perform M-GWAS where the multi-trait $\chi ^{2}$ was calculated using Eq. (2). Then, the $\chi ^{2}$ values were converted to *P*-values with 16 degrees of freedom.
3. The most significant variant (*P* < 10^–5^) from M-GWAS (excluding any previously selected most significant variants) was identified on each chromosome. The LD between the most significant variant and every other significant variant on the same chromosome was then tested using the PLINK v1.9 software [29]. If the LD between the most significant variant and any other significant variant that exceeded an r^2^ of 0.1, these other variants were considered as potentially tagging the same causal variant and hence were not eligible for selection. The remaining next most significant variant per chromosome (in LD, i.e. r^2^ ≤ 0.1 with the most significant variant) was then selected, and again LD between this and all remaining variants on the same chromosome was tested to determine if, in this cycle, there were any other remaining significant variants that were independent (r^2^ ≤ 0.1). This selection of the most significant independent QTL variants continued for each chromosome until no further significant variants representing independent QTL were eligible for selection in this cycle.
4. Then, each of the 16 single-trait GWAS was re-run per chromosome while jointly fitting the selected sets of independent significant QTL variants and conditionally re-testing all remaining variants. If there were no significant variants selected on a particular chromosome, then no conditional single-trait GWAS was required for this chromosome for any trait.
5. The newly derived t-values from each conditional single trait GWAS except for the significant variants selected from previous cycle(s) were used to recalculate the M-GWAS chi-squared statistic (Eq. (2)). The original t-values were used for the variants selected from previous cycle(s) that were fitted conditionally in the single-trait GWAS.
6. For the next cycle (and any following), the above steps 3 to 5 were repeated until no more variants were significant in the CM-GWAS.

Having identified all independent significant variants from the CM-GWAS, we then created a secondary expanded list that included all the significant M-GWAS variants (*P* < 10^–5^) in strong LD (r^2^ > 0.80) with the selected independent variants. We did this because it is possible that the most significant imputed sequence variant is in strong LD with the actual causal variant but the latter may show a slightly lower significance [13]. The nearest genes to each of these variant positions on the reference genome OAR3.1 were identified from UCSC Genome Bioinformatics (http://genome.ucsc.edu/) and Ensembl (www.ensembl.org/biomart/).

The power of QTL detection in the M-GWAS and the single-trait GWAS was investigated by comparing the FDR in each analysis [28]. Also, Q–Q plots were generated for all traits and the genomic inflation factor (lambda) for each trait was calculated to check if there was any inflation of observed chi-squared statistic due to population structure.

## Results

### Sequence GWAS

#### Single-trait GWAS

GWAS were performed for 16 wool quantity and quality traits (measured as yearling and adults) as well as breech wool cover and breech skin wrinkle scores for up to 7218 pure Merinos that had imputed genotypes for ~ 30 million WGS variants (Table 1). Estimates of the FDR for significant levels *P* < 10^–5^ and *P* < 10^–6^ varied between traits (Table 2). At *P* < 10^–6^, ten traits had more than 300 significant variants with an FDR lower than 9.1%, while at *P* < 10^–5^ FDR was generally higher, ranging from 9 to 51%, excluding mean fibre diameter at adult age (afd) and breech cover (ebcov). An alternative presentation of the results to the calculation of FDR is a Q–Q plot shown for each trait (see Additional file 1: Fig. S1). In addition, there was no indication of inflation of the test statistic due to population structure because the genomic inflation factor lambda was very close to 1 for all traits (see Additional file 2: Table S1). This is expected because population structure has been captured in our analysis by fitting both a random Merino strain effect and a genomic relationship matrix (see the model used in single-trait GWAS in “Methods” section).

**Table 2:** Number of significant variants (N) (*P* < 10^–5^ and *P* < 10^–6^) and their false discovery rates (FDR, %) for each trait from the single-trait GWAS and multi-trait GWAS

Trait^a^	*P* < 10^–5^		*P* < 10^–6^	
	N	FDR	N	FDR^b^
ygfw	1891	15.1	716	4.0
agfw	2164	13.2	910	3.1
ycfw	1145	24.8	606	4.7
acfw	1153	24.8	274	10.4
ysl	2182	13.1	1166	2.4
asl	753	37.9	103	27.7
yfd	1340	21.2	314	9.1
afd	312	91.4	16	
ydcv	3162	9.0	1722	1.7
adcv	1301	21.9	493	5.8
ycuv	682	41.8	145	19.7
acuv	562	50.8	183	15.6
yss	2516	11.3	1547	1.8
ass	1611	17.7	911	3.1
ebwr	1174	24.3	518	5.5
ebcov	354	81.0	14	
M-GWAS	9879	2.9	7431	0.4

*N* number of significant variants, *M-GWAS* multi-trait GWAS

^a^Trait names are as defined in Table 1

^b^FDR in empty cells are either not available or higher than 100%

**Fig. 2:**
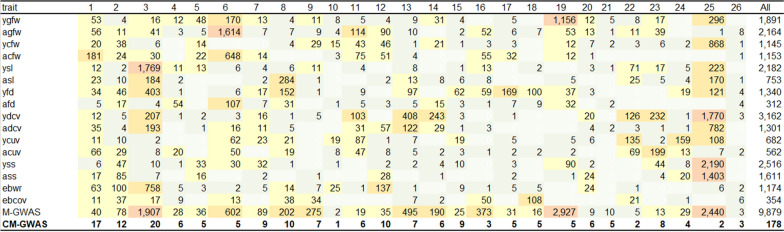
Number of significant (*P* < 10^−5^) variants for individual traits at yearling (y) and adult (a) ages from single-trait GWAS and multi-trait-GWAS and a total number of QTL identified on each of the 26 ovine chromosomes (OAR)

Figure 2 shows the number of significant variants (*P* < 10^–5^) per chromosome for each trait. Many significant variants (> 100) with pleiotropic effects were identified on *Ovis aries* (OAR) chromosome OAR3, 6, 8, 11, 22, 23 and 25. In addition, other clusters of variants that appear to affect only a single trait [e.g. variants on OAR17 for mean fibre diameter at yearling age (yfd)] were found. In a few cases, we observed regions with clusters of highly significant variants that appeared for traits measured at yearling but not for traits measured at adult age and vice versa. For example, over 600 significant variants were found on OAR6 for clean fleece weight at adult age (acfw), but none for clean fleece weight at yearling age (ycfw), whereas significant variants were observed on OAR25 for greasy fleece weight at yearling age (ygfw) and ycfw, but not for greasy fleece weight at adult age (agfw) and acfw. To demonstrate that this was not due to different numbers of animals being recorded and analysed at yearling and adult ages for greasy fleece weight, we re-analysed only the 4222 overlapping phenotypes available at both ages. A similar pattern of significance regions was still observed (see Additional file 3: Fig. S2).

#### Multi-trait GWAS (M-GWAS and CM-GWAS)

**Fig. 3:**
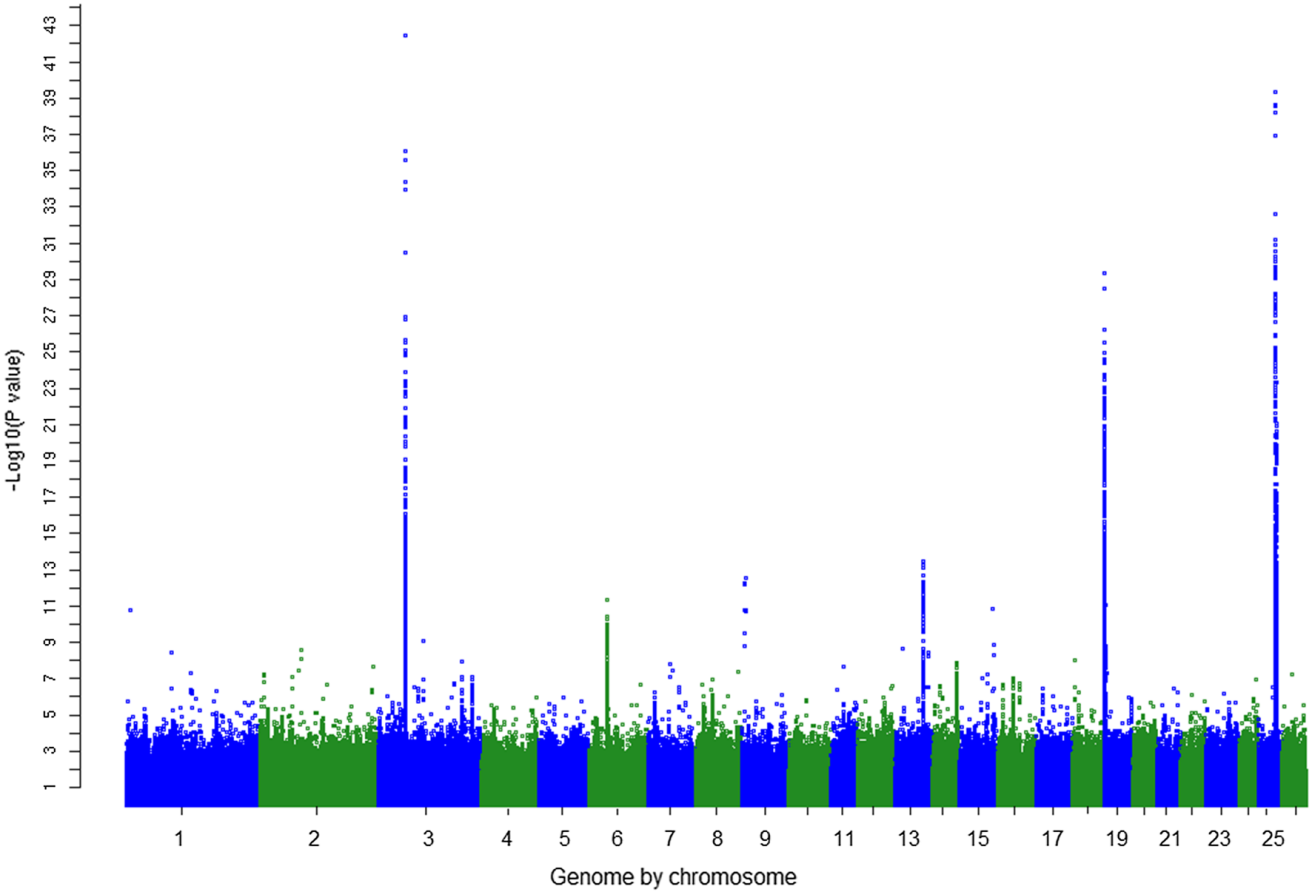
Manhattan plot of multi-trait meta-analysis (M-GWAS) before analysis of the conditional multi-trait GWAS (CM-GWAS) model. The −log_10_(*P*-values) of the multi-trait test on the y axis were calculated using SNP effects from the single-trait GWAS for 16 wool traits and genome positions on the 26 ovine autosomes are on the x axis

In the M-GWAS that combined the single-trait GWAS results, there were 9879 and 7431 significant variants at thresholds of *P* < 10^–5^ and *P* < 10^–6^, respectively. This corresponded to an FDR of 2.9 and 0.4%, respectively, which was lower than for any individual trait tested in the single-trait GWAS (Table 2). Many highly significant variants from the M-GWAS were found within narrow regions on OAR3, 6, 9, 13, 14, 16, 19 and 25 (Fig. 3). Within these clusters, many of the variants were in strong LD, therefore, to be able to identify independent putative causal variants we extended the published M-GWAS method to include a CM-GWAS. We selected only the most significant (‘top’) variants from each cycle of the CM-GWAS (*P* < 10^–5^; LD r^2^ < 0.10 with other ‘top’ variants) and identified 178 independent putative causal variants across the genome (see Fig. 2 for number per chromosome). These 178 variants were separated from each other by at least 127 kb: the LD regions, variant effects (signed *t*-values > 2 or < − 2), and the genes closest to these variants are presented in (see Additional file 2: Table S2). In total, 20 independent variants (out 178 variants in Additional file 2: Table S2) were located on OAR3, while only two remained on OAR25. All 178 significant CM-GWAS variants are putatively causal and most of them showed pleiotropic effects on the wool traits as would be expected from this analytical approach.

In addition, the list of 178 significant CM-GWAS variants was expanded to include other significant variants from the standard M-GWAS (*P* < 10^–5^), which showed strong LD (r^2^ > 0.80) with these 178 top variants, because the most significant imputed variant may not always be the causal variant. This resulted in 1510 variants and we annotated the nearest genes located within 100 kb on either side of each variant (see Additional file 2: Table S3). Thirty-one of the 178 independent variants were each in strong LD with 5 to 283 other significant variants. The length of these 31 LD regions ranged from 0.86 to 733 kb (see Additional file 2: Table S2). Many of the 178 CM-GWAS significant variants differed in some of their pleiotropic trait associations (see Additional file 2: Table S2), which suggests that the putative causal variants influence a range of physiological pathways that modify the expression of these traits.

### Putative causal variants and candidate genes

The largest number of highly significant variants in the M-GWAS (Fig. 2) was found within narrow regions on OAR3, 6, 13, 19 and 25 (Fig. 3) and (see Additional file 2: Table S2). In each of these regions, the CM-GWAS identified the most significant single independent putative causal variant as detailed below.

#### Variant OAR3:58,729,692

This putative causal variant (CM-GWAS *P* = 3.2 × 10^–43^, MAF = 0.06) was located within an intronic region of the *THNSL2* gene (*threonine synthase like 2*) and lies approximately 257 kb upstream of the *FOXI3* gene (58,986,757 to 58,990,671 bp)*.* The alternative allele was associated with almost every trait: increased fleece weight, fibre diameter coefficient of variation, breech wrinkle and breech coverage, as well as decreased staple length, mean fibre diameter, mean fibre curvature and staple strength. Although this variant was not in very strong LD (r^2^ > 0.80) with any other significant M-GWAS variants, 627 significant variants (M-GWAS: *P* < 10^–5^) were in moderate LD (0.10 < r^2^ < 0.74) with this variant within the 58.5–60.8 Mb region, encompassing the *FOXI3* gene.

#### Variant OAR25:35,305,108

This variant (CM-GWAS *P* = 4.8 × 10^–40^, MAF = 0.11) falls within an intron of the *MAT1A* gene (*methionine adenosyltransferase 1A*) and was in strong LD (r^2^ > 0.80) with 117 other significant variants. One of the variants in strong LD was a missense variant in *MAT1A* and was the third most significant variant (2.3 × 10^–39^). The alternative allele of OAR25:35,305,108 was associated with increased fleece weight, staple length, mean fibre diameter and staple strength, as well as decreased fibre diameter coefficient of variation and mean fibre curvature. The *t*-values (effect/SE) were consistently larger for the yearling traits than for the same traits measured in adults. This is probably due to larger numbers of animals with yearling age records. There was no significant effect of this variant for breech wrinkle or breech cover. Of the 2440 significant variants on OAR25 in the M-GWAS, only one other variant (OAR25:30,581,246) remained significant (*P* < 10^–5^) after jointly fitting OAR25:35,305,108 in the CM-GWAS model.

#### Variant OAR19:840,732

This is a missense variant (CM-GWAS *P* = 4.4 × 10^–30^, MAF = 0.12) in the *EGFR* gene (*epithelial growth factor receptor*) and was in strong LD (r^2^ > 0.8) with 246 other significant variants (M-GWAS: *P* < 10^–5^). The alternative allele was associated with increased greasy fleece weight and staple length, as well as decreased fibre diameter coefficient of variation and mean fibre curvature. There were no significant effects on clean fleece weight, staple strength, breech wrinkle or breech cover.

#### Variant OAR13:62,835,771

This is an intergenic variant (CM-GWAS *P* = 3.3 × 10^–14^, MAF = 0.12) located 16 kb from the *RALY* (*RALY heterogeneous nuclear ribonucleoprotein*) gene and 71 kb from the *EIF2S2* (*eukaryotic translation initiation factor 2 subunit beta*) gene. It was in strong LD (r^2^ > 0.8) with 283 other significant variants (M-GWAS: *P* < 10^–5^) spanning a region encompassing both the *RALY* and *EIF2S2* genes. The alternative allele was associated with increased fleece weight, staple length, fibre diameter, and fibre diameter coefficient of variation, as well as decreased mean fibre curvature, staple strength, and breech wrinkle.

#### Variant OAR6:37,676,407

This is an intergenic variant (CM-GWAS *P* = 4.9 × 10^–12^, MAF = 0.39) and was in strong LD (r^2^ > 0.8) with 249 other significant variants (M-GWAS: *P* < 10^–5^) spanning 0.7 Mb, located between 36.97 and 37.71 Mb. Among the M-GWAS significant variants, 12 variants were within the *LCORL* gene (*ligand dependent nuclear receptor corepressor like*) and 24 in the *NCAPG* gene (*non-SMC condensin I complex subunit G*). Although the variants within these genes included three missense variants, they showed a somewhat lower significance level (7.9 × 10^–8^) than the OAR6:37,676,407 variant. The latter variant was associated with increased fleece weight and fibre diameter coefficient of variation as well as decreased mean fibre diameter and staple length.

#### Additional CM-GWAS candidate genes

While it is not possible to discuss all of the 178 candidate genes and variants (see Additional file 2: Table S2), we highlight several additional variants that showed strong pleiotropy across both fibre and skin wrinkle traits and were identified near or within interesting candidate genes: *ALX4, EIF2AK2*, *ESRP1*, *HAS2*, *MC5R* and *MX2* (Table 3).

**Table 3:** List of putative causal variants with the signed significant t-values (|t| ≥ 2.0) across wool traits at yearling and adult ages

OAR pos. (bp) for most significant SNP	Annotation class for the most significant variant	M-GWAS Pval	ygfw	agfw	ycfw	acfw	ysl	asl	yfd	afd	ydcv	adcv	ycuv	acuv	yss	ass	ebwr	ebcov	Flanking genes^a^
Chr3:58,729,692	Intronic	3.21E−43		4.6		3.9	− 10.9	− 5.5	− 6.7	− 3.9	3.3	5.1		− 3.2	− 2.3	− 4.2	6.9	3.5	*THNSL2*^0^
Chr25:35,305,108	Intronic	4.85E−40	5.1	2.9	6.7	3.3	4.6	4.6	4.4	2.8	− 9.6	− 6.8	− 4.5	− 3	10.4	8.8			*MAT1A*^0^
Chr19:840,732	Missense	4.44E−30	4.9	4.1				2			− 2.5		− 2.1	− 2.6					*EGFR*^0^
Chr13:62,835,771	Intergenic	3.30E−14		4	2	3.8	4	3.1	4.7	3.4	6.2	4.7		− 2.2	− 2.5		− 2.5		*RALY*^16^ *EIF2S2*^71^
Chr6:37,676,407	intergenic	4.87E-12	3.9	6.2	3.6	5.2	− 2.6			− 3.1	3.5	2.1							*LCORL*^224^
Chr15:72,567,959	intronic	5.92E-09					2.9		6.6	3.8							− 3.7		*ALX4*^0^
Chr3:86,858,557	Intronic	1.29E−07		2	2.3						5.1	3.7					− 2.7		*EIF2AK2*^0^
Chr9:82,254,047	Intronic	8.29E−07						3.8			3.9		− 2.8	− 2.1	− 2.7		− 2.9		*ESRP1*^0^
Chr23:43,896,965	Upstream	3.07E−06							− 3.3	− 2.9	5.4	3.4			− 3.3	− 2.5	3.6		*MC2R*^2.2^
																			*MC5R*^27^
Chr9:29,973,331	Intergenic	4.59E−06	3.2		5.2		3.4	2.8						− 2.1			− 2.4		*ZHX2*^327^
																			*HAS2*^970^
Chr3:58,952,741	Intergenic	3.41E−06					2.8		4.2	3.4	−2.4		2			3.9	− 4.6	− 2.1	*FOXI3*^34^
																			*THNSL2*^213^
Chr1:259,689,936	Intronic	1.97E−06				2.2	2	3.1		3.1			− 5.8	− 3.9		2.7	− 2.1		^b^*MX2*^0^

^a^Number in superscript shows the distance to each gene in kb

^b^*MX2* lies very close to LOC101102526, which is a *keratin-associated protein 10–12-like* gene

## Discussion

To our knowledge, this is the first study to undertake a large-scale GWAS using whole-genome sequence genotypes for wool traits and skin wrinkle in sheep. Our results fine-mapped 178 significant putative causal variants and candidate genes for wool traits and breech skin wrinkle in independent QTL regions from over 30 million whole-genome sequence variants. Independent QTL regions were identified by applying our CM-GWAS that cycled through multiple rounds of single- and multi-trait GWAS to conditionally identify the most significant variants in the multi-trait GWAS. The selection of the most significant variants was also conditional on a very stringent pairwise LD (r^2^ < 0.1) with other significant variants. The CM-GWAS could be applied to any species measured for a set of traits. The threshold *P* value for the multi-trait will depend on the power of the analysis, and we also recommend a stringent LD threshold to determine QTL independence. We used LD r^2^ of 0.1 so that in each cycle there was little risk of selecting more than a single variant from each QTL, particularly for variants that had relatively large effects. Then, if a variant in LD r^2^ > 0.1 remained significant in the following cycle after conditional analysis, then the variant could be selected in that cycle.

This relatively conservative approach enabled identification of large and/or highly pleiotropic variant effects for wool and breech wrinkle traits that may be the causal variants or be in very strong LD with causal variants. Imperfect sequence imputation may result in a causal variant having a lower *P* -value than one or more other variants in strong LD with it [13]: to mitigate this, we annotated other significant variants that were in strong LD (r^2^ > 0.8) with the most significant putative causal variant (see Additional file 2: Table S3). Indeed, among the five most significant putative causal variants, four were in strong LD with over 100 other significant variants (Chr6:37,676,407; Chr19:840,732; Chr25:35,305,108; and Chr13:62,835,771). This strong LD spanned over 150 kb suggesting that there has been strong selection across these regions.

The direction of the various trait effects for pleiotropic mutations are of great interest to the wool industry because they may decrease or increase overall wool value. In general, the most desirable wool attributes are increased clean fleece weight (cfw) and staple strength (ss) with decreased fibre diameter (fd), and coefficient of variation in fibre diameter (dcv). In addition, reduced breech cover (ebcov) and reduced breech skin wrinkle (ebwr) would help reduce the incidence of breech flystrike that has a large economic impact on the Merino industry. Among these traits, the strongest economic indicators are for cfw and fd, however there is an unfavorable positive genetic correlation between fd and cfw in Merinos (~ 0.3; [30], which suggests that the more common pattern of pleiotropy would be that where effects follow the same direction. Indeed, among the 178 variants, 24 showed pleiotropy for cfw and fd, and of these, 15 were in the same direction, while only nine showed effects in opposing directions (see Additional file 2: Table S2). Of the 68 variants with an effect on adult cfw, for all but one, the alternative (i.e. non-reference) allele was associated with increased fleece weight. This is perhaps somewhat expected given that the OAR3.1 reference genome was obtained from a Texel sheep: a breed with much lower fleece weights than Merinos. There is also an undesirable positive genetic correlation between breech wrinkle and fleece weight, as well as an undesirable negative genetic correlation between wrinkle and fibre diameter [4]. Again, among the 178 most significant CM-GWAS variants, those that decreased wrinkle also decreased fleece weight (and vice versa) and/or increased fibre diameter (and vice versa).

The variant on OAR6 (37,676,407 bp) was in a strong LD region that is better known for associations with body size in a range of mammalian species including humans [11, 31–35] although evidence for the exact mechanism and causal variants remain elusive. This most significant variant in our study was intergenic (*P* = 5 × 10^–12^) and was in strong LD with several missense variants (among other significant variants) in the *NCAPG* and *LCORL* genes (*P* = 8 × 10^–8^) that have been reported to be located within a signature of selection observed between Merino and Churra sheep [36]. However, it is not clear what effect the genes in this region may have on wool production.

A second variant (OAR19:840,732) showing an extended region of strong LD was a missense mutation in the *EGFR* gene. The protein encoded by *EGFR* is a cell surface protein that binds to epidermal growth factor (EGF) and is well documented as playing a key role in skin and hair follicle development, as reviewed by [26]. Previously, a point mutation in the *EGFR* gene has been demonstrated to be responsible for the mouse “wavy hair” phenotype [37]. Furthermore, mice that harbor a targeted disruption of the *epidermal growth factor receptor* (*EGFR*) allele exhibit a severely disorganized hair follicle phenotype and fuzzy coat [38]. In our study, the missense putative causal variant was associated with fibre curvature, staple length and greasy fleece weight but, interestingly, had no effect on clean fleece weight. This suggests that the increase in greasy fleece weight is either due to some modification of the sebaceous gland output of suint, or that the modified fibre characteristics resulted in the fleece holding more dust and/or suint. Related to the effect of *EGFR* on wool traits was a different significant variant on OAR11 (11,374,041 bp) located in an intergenic region showing moderate LD and 11 kb from the *PIK3R1* gene that has a GO term: “epidermal growth factor receptor signalling pathway”. This variant had the strongest association with staple length and fibre curvature.

A third putative causal variant (Chr25:35,305,108) in a region of strong LD was in an intron of the *MAT1A* gene. This gene codes for a key enzyme (methionine adenosyltransferase 1A) in the pathway that converts methionine to cysteine. These two sulphur amino acids have been shown to be rate limiting for wool production in sheep [39], such that supplementing sheep (per abomasum to bypass the rumen) with these amino acids resulted in increased wool weight, staple strength and fibre diameter. Interestingly, these effects are in keeping with the pleiotropic effects observed for our putative causal variant. Furthermore, the strongest effects of this mutation were observed for the yearling rather than the adult traits, which suggests that this may be due to stronger competition for sulphur amino acids in young growing sheep. There was a missense variant in strong LD (r^2^ > 0.8) within the same gene, OAR25:35,301,334 (see Additional file 2: Table S3), which lies just 3774 bp away. It is plausible that this missense mutation maybe the true causal variant because it was the third most significant with a *P* value (2.3 × 10^–39^) very close to that of the most significant variant (4.8 × 10^–40^). To our knowledge there is only one other report of a QTL for wool traits close to the *MAT1A* gene and, in that study, a subset of the same animals as ours was analysed but only with high-density marker genotypes [7].

The fourth highly significant pleiotropic variant in strong LD with many surrounding variants was an intergenic variant on OAR13 (62,835,771 bp) and the LD region spanned variants in and close to the *RALY* and *EIF2S2* genes. While the *RALY* gene has been reported to show a strong signature of selection between sheep breeds [40], it is not documented as having any reported effects on fibre production. However, the *EIFS2S* gene has been reported to play a role in a major mutation that discriminates between the ancestral sheep with a coarse longer hair-like coat from the modern domestic descendants with a finer and shorter wool coat [41]. These authors describe the mutation resulting in the “woolly” coat as an antisense *EIF2S2* retrogene (called *asEIF2S2*) that is inserted into the 3′ UTR of the *IRF2BP2* gene. The hybrid mRNA that is transcribed from the disrupted *IRF2BP2* gene was demonstrated to affect the expression of both the EIF2S2 and IRF2BP2 mRNA. The pleiotropic effects of our putative causal variant in our study are in keeping with these authors’ observations, i.e., increased fleece weight, staple length and fibre diameter as well as reduced curvature. It is possible that there is a causal variant among the significant variants that we detected, which alters the expression or the transcript of the *EIFS2S* gene. The strong LD across this region is potentially accentuated because *EIFS2S* lies close to a large 149-kb duplication encompassing the *ASIP* gene that results in the dominant white colour of Merinos [42]. Interestingly we found an intronic variant in the related *EIF2AK2* gene which like *EIF2S2* also had the strongest effect on the coefficient of fibre diameter and breech wrinkle (Table 3). Similarly, a variant in the *MX2* gene (Table 3) was very strongly associated with fibre curvature but also breech wrinkle and a number of other traits. The *MX2* gene has a GO term: “regulation of cell cycle” and has been found to be significantly differentially expressed in the skin of super fine Merino sheep compared to a coarse wool sheep breed [43].

The most highly significant variant in our CM-GWAS on OAR3 (58,729,692 bp) showed only moderate to low LD with other significant variants (0.10 < r^2^ < 0.74) and had a low MAF (0.06), which indicates that it may be a relatively recent mutation. We observed that after fitting this variant in the conditional GWAS all 1644 significant variants in the 57.5–61.2 Mb region on OAR3 (except 11 variants) became no longer significant, and thus this variant seems unlikely to be spurious. It is of course also possible that the imputation in this region is less accurate than average for some reason, and that this has affected the LD pattern in the region. While this mutation was associated with a favourable effect for the sheep industry, i.e. increasing wool weight and decreasing fibre diameter, this is also accompanied by an unfavourable increase in breech wrinkle as well as breech wool cover. Breech wrinkle and breech cover are indicator traits associated with breech flystrike because deep wrinkles (characteristic of some Merinos) combined with wool cover in the breech area increases the risk of urine and faecal staining. This provides a moist environment for flystrike and therefore breech wrinkle and cover traits are used by some breeders to select for resistance to breech flystrike. About 40 variants (out of 178) had a significant effect on breech wrinkle and most of these had an antagonistic effect either with mean fibre diameter and/or fleece weight (e.g. decreased wrinkle and increased fibre diameter). The putative causal variant on OAR3 (58,729,692 bp) lies in an intronic region of the *THNSL2* gene, which to our knowledge has not been previously reported as being associated with wool/fibre characteristics or skin wrinkle. Furthermore, the GO terms associated with this gene and extensive literature searches suggest no obvious role for *THNSL2* in the traits analysed. However, the closest flanking protein coding genes are *FABP1* (*fatty acid binding protein 1*) and *FOXI3* (*forkhead box I3*) with the latter lying ~ 250 kb downstream. Previously, a 7-bp duplication in an exon of *FOXI3* (causing a premature stop codon) has been reported to cause a hairless phenotype in some dog breeds [44]. Our putative causal mutation (OAR3: 58,729,692) was associated with much shorter and finer hair, but also with the largest effect observed for skin wrinkle. We also observed another apparently independent putative causal variant closest to the *FOXI3* gene that was also strongly associated with breech wrinkle (58,952,741 bp: Table 3). Although there were no previously documented reports of associations between *FOXI3* and skin wrinkle, the *FOXI3* gene is associated with the GO term “cell differentiation” (GO:0030154). In the Sheep Tissue Atlas [45] (see https://www.ncbi.nlm.nih.gov/gene/?term=FOXI3++sheep), the highest tissue expression of *FOXI3* was in skin. The family of *FOX* genes encode transcription factors that are involved in functions such as: cell differentiation and proliferation [46]. Interestingly, another forkhead box protein gene, *FOXN1*, also highly expressed in the skin was reported to cause hypotrichosis (abnormal hair growth) and highly wrinkled skin in Birman cats [47]. The putative causal variant in the *THNSL2* gene also lies within a long non-coding RNA (within this gene), so it could even be possible that this plays some regulatory role on the adjacent *FOXI3* gene. We found two additional significant pleiotropic intergenic variants close to the *FOXK1* and *FOXB1* genes.

The significant variant with an undesirable effect on fibre diameter was in the *ALX4* gene which is known to have a role in hair follicle growth and cycling [48, 49]. Kijas et al. [50] also found strong evidence for a sheep selection signature in the region around the *ALX4* gene. A breech wrinkle variant that was strongly associated with staple length but did not associate with either fleece weight or fibre diameter was located in the *ESRP1* gene (*epithelial splicing regulatory protein 1*). This gene has a GO term “fibroblast growth factor receptor signalling pathway”. This is very interesting because the *FGF5* (*fibroblast growth factor 5*) gene is known to act as an inhibitor of anagen phase of hair cycle that has been demonstrated to have a direct impact on fibre growth across numerous species [51, 52]. The *ESRP1* gene regulates alternative splicing events in epithelial cells of the epidermis, and double knockouts of the *ESRP1* and *ESRP2* in mice impacted hair follicle maturation and epidermal thickness [53]. *ESRP1* is also very highly expressed in sheep skin (Sheep Atlas displayed on NCBI Gene information: [45]). Incidentally, we located a significant CM-GWAS variant at Chr16:31,054,761 that lies close to the *FGF10* gene and another one (Chr10:35,310,820) in a gene desert, but near *FGF9*, and both were associated with staple length as well as some other traits. We found two significant variants that reduced breech cover while not having very strong effects on other traits: one (Chr1:197,166,904) in the *LPP* gene (*LIM domain containing preferred translocation partner in lipoma*) and the second (Chr12:71,641,048) was just upstream of the *IRF6* (*interferon regulatory factor*) gene. The *IRF6* gene is very highly expressed in skin and is linked to the GO terms: keratinocyte proliferation and differentiation, cell cycle arrest. These findings may be useful for future research to help reduce breech wrinkle and wool cover and to lower the incidence of flystrike.

A very interesting significant variant that was associated with reduced breech wrinkle, increased fleece weight and staple length, but was not associated with fibre diameter (Table 3) and lies in a gene desert on Chr9 (29,973,331 bp) downstream from the *ZHX2* gene (*zinc fingers and homeoboxes 2*) and upstream of the *HAS2* gene (*hyaluronan synthase 2*; see Table 3). In the Shar-Pei dog breed a large ~ 16-kb duplication approximately 0.35 Mb upstream of the *HAS2* gene has been found to enhance the expression of the *HAS2* gene causing the characteristic thick skin folds particularly around the head of this breed [54].

There are few GWAS reports on sheep wool traits for us to compare our results to. In a single-trait GWAS with 50k SNP array genotypes in Chinese Merino sheep, Wang et al. [55] identified 28 SNPs located within 12 genes that affect fibre diameter, fibre diameter coefficient of variance, and crimp. However, we did not find any significant variants (*P* < 10^–5^) within these genes, which is perhaps not surprising given the low density of their genotypes and smaller number of animals studied. The only other report was our own study using a subset of the animals in this study but using only a high-density SNP array, therefore we have not included a comparison of those previous results here.

It is of interest to understand if most candidate genes associated with wool traits in sheep have already been identified in other mammalian studies. Thus, we extended our investigation further by compiling a set of genes (Published-Gene set) that were previously reported in the literature as associated with wool, fur, fibre or hair characteristics across humans, mice and other mammals (see Additional file 2: Table S4), and fine-mapped and estimated the variance explained by a subset of sequence variants in and around several hundred genes associated with hair, fur or wool fibre characteristics across different mammals (for details see Additional file 4). Interestingly, the Published-Gene set explained 6 to 17% more genomic variance than randomly selected sets (Random-Gene sets) for the traits related with fibre quality characteristics, but there was no clear difference between Random-Gene and Published-Gene sets for fleece weight, breech wrinkle or breech cover traits (see Additional file 4). Although there was some overlap between the CM-GWAS candidate genes and the genes previously reported in the literature as associated with mammalian fibre traits, many were not included in this gene list, which indicates that our study brings new knowledge to this field.

## Conclusions

To our knowledge, this is the first multi-trait meta GWAS study of wool traits using imputed sequence data in sheep. Our findings confirm the high polygenic and pleiotropic nature of the variants that affect wool traits as well as breech wrinkle and cover. Our CM-GWAS meta-analysis enabled the detection of 178 independent QTL regions and identified putative candidate variants and genes that may affect these traits such as a missense variant in *EGFR* (a gene known to affect fur growth in mammals). Novel candidate genes identified here included the *MAT1A* gene that encodes an enzyme in the sulphur metabolism pathway critical to production of wool proteins in high-producing Merino sheep, and the *ESRP1* gene. We also discovered a significant breech wrinkle variant upstream of the *HAS2* gene, which is known to cause the exaggerated skin folds of the Shar-Pei dog breed. These findings and additional functional annotation will further enhance our ability to exploit sequence mutations to improve wool and welfare traits in sheep.

## Supplementary Information

**Additional file 1: Figure S1.** Quantile–quantile plot of P-values from single-SNP genome wide association study (GWAS) for each of the 16 traits studied (dark orange) and from multi-trait meta GWAS (dark magenta). Observed and expected *P*-values would fall on the light blue line if there was no association.

**Additional file 2: Table S1.** Inflation factor (lambda) for each trait. The inflation of observed chi-squared statistic due to population structure was checked. **Table S2.** Annotation of pleiotropic effects on individual traits for 178 putative causal variants identified from the multi-trait conditional GWAS. The annotation includes positions, functional classification, rs ID where available and signed significant t-values (|t| > 1.96) were provided for 178 variants across the 16 traits at two ages. Gene names and distances from the nearest genes are also provided if variants are located in or within 100 kb of the gene. **Table S3.** Annotation of 1510 sequence variants in strong LD with the 178 most significant variants from multi-trait conditional GWAS. The annotation includes the positions, rs ID where available, functional classification and P values of multi-trait $\chi ^{2}$ statistic. The names of the nearest genes are also provided if variants are located in or within 100 kb of the gene. **Table S4.** List of 453 published genes previously associated with fibre characteristics in mammals (“Published-Gene set”) used to identify sequence variants to be includes in BayesR and GBLUP analysis. Ensembl identification, name of genes and gene location (chromosome number, start and end position in bp).

**Additional file 3: Figure S2.** Manhattan plot of single-trait GWAS for greasy fleece weight at yearling (a) and adult (b) ages using all animals and at yearling (c) and adult (d) ages using the same animals. The red points represent significant variants at *P* < 10^–6^.

**Additional file 4.** Analyses of variants located around previously reported wool and hair genes. A set of variants in and close to 453 previously reported genes associated with wool/fur/hair traits across mammals (Published-Gene set) were identified including 1619 coding variants and 11,712 variants up- and down-stream of the genes. A BayesR fine-mapping analysis identified which of these variants were most associated with wool traits and a GBLUP tested the proportion of genetic variance that the sequence variant set explained for each of the wool traits.

## Data Availability

All the SheepGenomesDB Run2 sequence data used for imputation to sequence is freely available. A list of the 935 SRA Sample ID here: Brauning R, McWilliam S, Daetwyler H, McCulloch A, Clarke S. SheepGenomesDB run2—list of animals. figshare. Dataset. 2020. 10.6084/m9.figshare.11530665.v1.
