# Supplementary figures and images for: A conditional multi-trait sequence GWAS discovers pleiotropic candidate genes and variants for sheep wool, skin wrinkle and breech cover traits
Source: Genet Sel Evol. 2021 Jul 8;53:58. doi: 10.1186/s12711-021-00651-0 (PMC8268212; doi:10.1186/s12711-021-00651-0)

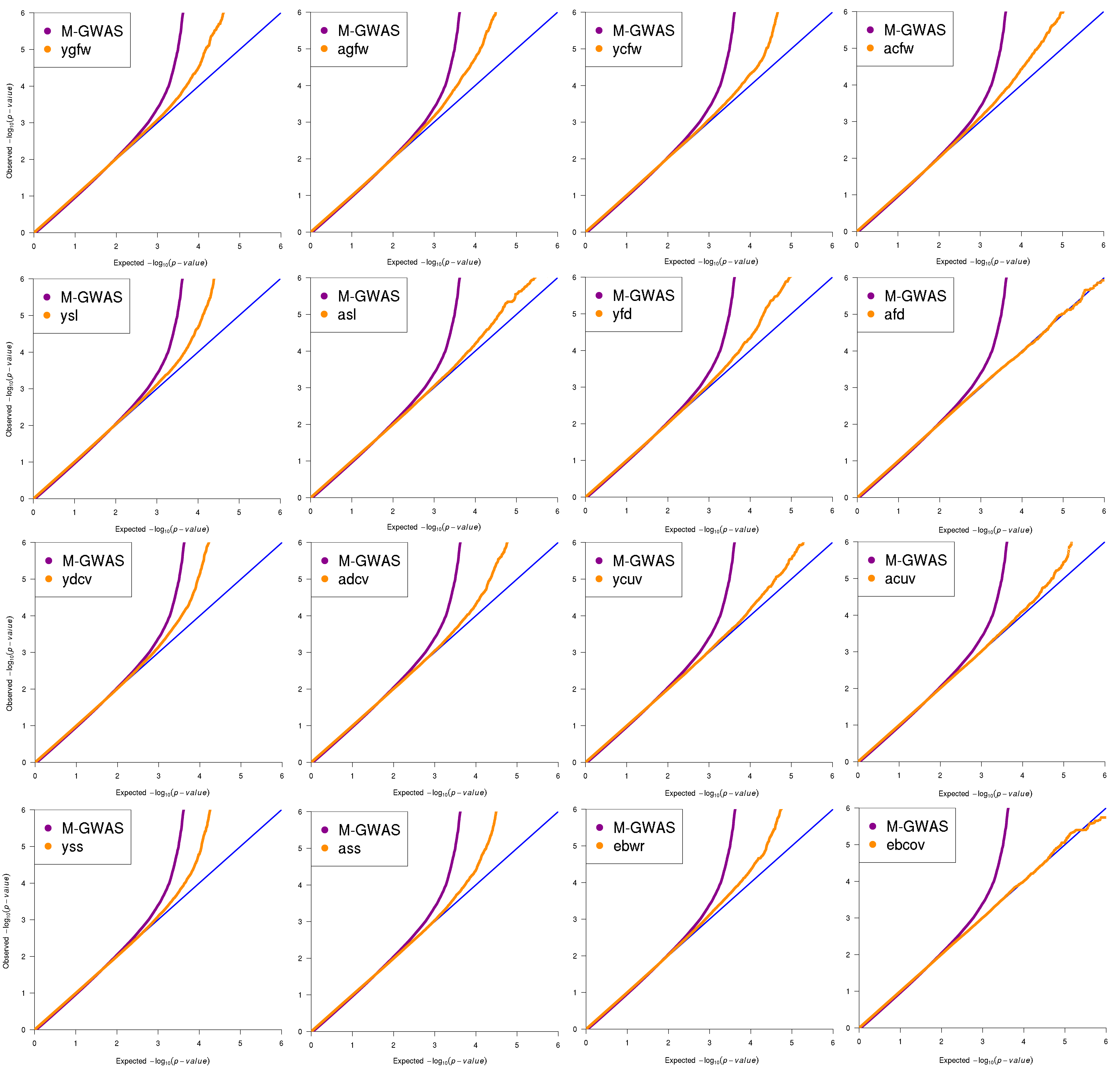

Supplement: Supplementary file 1 — Additional file 1: Figure S1. Quantile–quantile plot of P-values from single-SNP genome wide association study (GWAS) for each of the 16 traits studied (dark orange) and from multi-trait meta GWAS (dark magenta). Observed and expected P-values would fall on the light blue line if there was no association. [file 12711_2021_651_MOESM1_ESM.png]

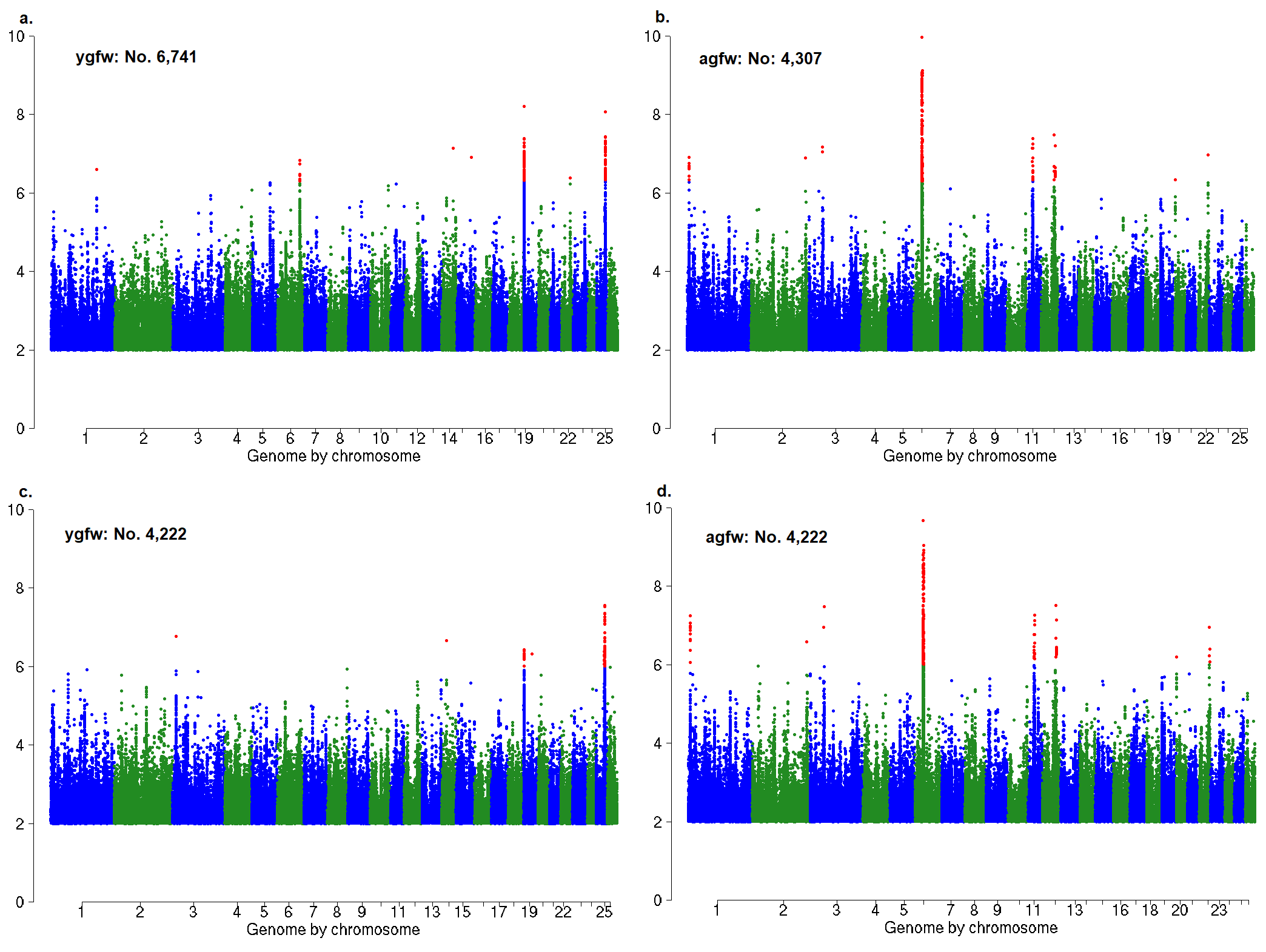

Supplement: Supplementary file 3 — Additional file 3: Figure S2. Manhattan plot of single-trait GWAS for greasy fleece weight at yearling (a) and adult (b) ages using all animals and at yearling (c) and adult (d) ages using the same animals. The red points represent significant variants at P < 10–6. [file 12711_2021_651_MOESM3_ESM.png]
