# Supplementary material for: A conditional multi-trait sequence GWAS discovers pleiotropic candidate genes and variants for sheep wool, skin wrinkle and breech cover traits
Source: Genet Sel Evol. 2021 Jul 8;53:58. doi: 10.1186/s12711-021-00651-0 (PMC8268212; doi:10.1186/s12711-021-00651-0)
Supplement: Supplementary file 4 — Additional file 4. Analyses of variants located around previously reported wool and hair genes. A set of variants in and close to 453 previously reported genes associated with wool/fur/hair traits across mammals (Published-Gene set) were identified including 1619 coding variants and 11,712 variants up- and down-stream of the genes. A BayesR fine-mapping analysis identified which of these variants were most associated with wool traits and a GBLUP tested the proportion of genetic variance that the sequence variant set explained for each of the wool traits. [file 12711_2021_651_MOESM4_ESM.docx]

**Previously reported wool and hair genes**

It is of interest to understand if most candidate genes associated with wool traits in sheep have already been identified through various mammalian studies. Using a set of variants in and around genes previously identified to affect fibre and hair traits in mammals, a BayesR analysis was used to fine-map the variants associated with the 16 wool traits used in our study. Also using this variant set or a random set, a GBLUP analysis demonstrated that these genes do explain some genetic variance in the wool traits used in our study.

**Methods**

We compiled a set of genes previously reported in the literature as associated with wool, fur, fibre or hair characteristics across humans, mice and other mammals. These genes were retrieved using the following links: OMIM (https://www.omim.org/), MGI (<http://www.informatics.jax.org/>), and OMIA (<http://omia.org/home/>). Additionally, we included candidate genes reported in [1-4]. Out of 607 genes, 486 genes (123 human, 255 mice and 124 from other mammals) were annotated in the ovine genome. In total, 453 genes were used for further analyses (after excluding genes on the *Ovine* X chromosome) and these are listed in [Additional file 1 Table S1]. An alternative approach to using GWAS to fine-map independent putative causal variants is to fit all variants simultaneously in the model using an approach such as BayesR [5]. However, this is computationally too slow with millions of variants, so we restricted the variant set to the Illumina Ovine 50K SNP panel plus sequence variants that were close to or within these genes previously reported in the literature

In each of the 453 genes we identified a total of 2,268 non-synonymous variants in coding regions as well as 40,210 potentially regulatory variants located up to 5,000 bp up- and down-stream. These variants were further filtered based on minor allele frequency (MAF < 0.005) and linkage disequilibrium (LD) pruning. The LD (r^2^) pruning was processed using PLINK software (V1.9, [6]) where one of each pair of SNP was removed if r^2^ was more than 0.85. In total, 13,331 variants were identified in this final set (referred to henceforth as “Published-Gene set”) consisted of: 1,619 coding variants and 11,712 in regulatory regions.

We used two analytical methods with the Published-Gene set: 1) GBLUP to estimate the genetic variance explained by variants in the Published-Gene set and 2) BayesR for fine mapping variants most associated with wool traits.

**GBLUP**

It is of interest to estimate genetic variance explained by variants identified in the regions of previously reported hair and wool genes (Published-Gene set), therefore we tested this using ASReml software [7]. The genetic variance explained by both the 50K SNP panel and the Published-Gene set was estimated by fitting the GRM for these two sets of genotypes separately in the following model:

**y** = **1_n_**µ + **Zg_1_** + **Zg_2_** + **e**

where **y** is a vector of phenotypes, **1**_n_ is a vector of ones, *μ* is the overall mean, **Z** is the design matrix allocating records to breeding values, $\mathbf{g}_{j}$(j=1 is for 50k SNP set and j=2 is for Published-Gene set) is a vector of genomic breeding values $\sim N(\boldsymbol{0}, \mathbf{G}_{j}\sigma_{g}^{2}$), where $\sigma_{g}^{2}$ is the genetic variance and $\mathbf{G}_{j}$is the GRM, and ***e*** is the vector of residuals (normally distributed with variance $\sigma_{e}^{2}$). As for all other analyses, variants were only included in the GRM provided their MAF was greater than 0.005 in the whole dataset. The phenotypes were pre-corrected for Merino strains (described in the GWAS section) before performing GBLUP.

To assess whether the variants in the Published-Gene set explained more variance than might be expected by chance, GBLUP was also performed on a further 10 independent Random variant sets, each consisting of coding and regulatory variants in 10 different randomly selected sets of 453 genes, after first removing the 453 hair/fleece genes and 2,110 other genes which were located within 5 kb of these 453 hair/fleece genes. The variants in the Random sets were also pruned for LD and MAF as for variants in the Published-Gene set.

**BayesR**

The BayesR method [5] was used to jointly fit the entire Published-Gene set of variants together with the standard Ovine Illumina 50k array genotypes. BayesR assumes that SNP effects are from a mixture of four normal distributions with the variance of each distribution equal to 0, 0.01%, 0.1% or 1% of the genetic variance. Gibbs sampling was used to sample from the posterior distributions of the parameters, running 40,000 iterations with 20,000 iterations of burn-in. The phenotypes were pre-corrected for Merino strains as described in the GBLUP analysis. The model fitted in the BayesR analysis was:

**y** = **1_n_**µ + **Wv** + **e**

where **1**_n_ is a vector of ones, *μ* is the overall mean, **W** is the design matrix of SNP genotypes (centered and standardised to variance =1) and **v** is the vector of SNP effects and **e** the vector of residuals.

Five Gibbs sampling chains were run for each trait to check for convergence across chains. The BayesR posterior probability of SNP having an effect on the trait was averaged across chains and used to identify SNPs that had the highest association with a trait.

**Results**

**Genetic variance explained by variants associated with the reported wool and hair genes**

We identified 13,331 sequence variants (‘Published-Gene set’) in coding and regulatory regions of previously reported genes with major effects on wool / hair / fur [Additional file 1 Table S1]. The variance explained by the Published-Gene set for each trait was estimated using a GBLUP model that simultaneously fitted two GRM: one constructed from the Ovine 50k SNP array genotypes and the other from the Published-Gene set sequence genotypes. This was contrasted with the average variance explained by 10 Random variant sets (mimicking the Published-Gene set but from randomly selected sets of genes). The proportion of genomic variance explained by the Published-Gene set compared to total genomic variance varied from 0% (ebcov) to 30 % (yss) (Table 1). Interestingly, the Published-Gene set explained 6 – 17% more genomic variance than Random sets for the traits involving fibre quality characteristics, but there was no clear difference between Random-Gene and Published-Gene sets for fleece weight, breech wrinkle or breech cover traits (Table 1). The genomic heritability estimates were moderate to high for most wool traits (except ebcov) and ranged from 0.26 (yss) to 0.69 (afd).

**BayesR to finemap variants and candidate genes**

We used BayesR to fine-map a subset of WGS variants in and around several hundred genes that were reported to have associations with hair, fur or wool fibre characteristics across different mammals. Across 14 traits (excluding ebwr and ebcov), 231 variants from the Published-Gene set had a posterior probability ($\mathrm{pp}$) higher than 0.25 of having a non-zero effect. These variants were within or close to 155 genes. Table 2 shows only the 14 variants with largest effects that had a posterior probability higher than 0.5 of affecting at least one trait (Table 2 also indicates pleiotropy for these variants, showing the posterior probability ≥ 0.2 for other traits). Out of the 14 variants in Table 2, seven were found to be significant at *P* < 10^-5^ in the M-GWAS and six of the related genes overlapped genes annotated for the most significant M-GWAS variants or for variants in strong LD: *ALX4*, *EGFR*, *EIF2S2*, *FOXI3*, *MAT1A* and *MC5R*. The trait pleiotropy for these candidate genes mirrored the single trait GWAS results. The BayesR analysis included variants close to *EIFS2S* (but not *RALY*) and found strong associations for two variants in and close to *EIFS2S* affecting length, fibre diameter and curvature (Table 2). The missense variant on OAR19 at 840732 bp in the *EGRF* gene was also the most significant variant in the CM-GWAS. Similarly, the missense variant in the *MAT1A* gene (OAR25:35301334) was the 3^rd^ most significant variant in the M-GWAS and was in very strong LD (r^2^ > 0.8) with the most significant M-GWAS variant (intronic) just 3,774 bp away. The most significant CM-GWAS variant was intronic and therefore could not overlap the BayesR analysis because intronic variants were not included in the BayesR analysis to reduce computational time. In both analyses the significant variant in the *ALX4* gene was strongly associated with reduced wrinkle but also strongly increased fibre diameter. The *ALX4* gene is known to be associated with hair follicle growth and cycling [8, 9]. Kijas et al. [10] also found strong evidence for a selection signature in the region around the *ALX4* gene.

**References**

1. Duverger O., Morasso MI. To grow or not to grow: hair morphogenesis and human genetic hair disorders. Semin Cell Dev Biol. 2014:https://dx.doi.org/10.1016%1012Fj.semcdb.2013.1012.1006.

2. Bolormaa S, Swan AA, Brown DJ, Hatcher S, Moghaddar N, van der Werf JH et al. Multiple-trait QTL mapping and genomic prediction for wool traits in sheep. Genet Sel Evol. 2017;49:62.

3. Schweizera J, Langbeinb L, Rogersa MA., H. W: Hair follicle-specific keratins and their diseases. Exp Cell Res 2007:DOI: 10.1016/j.yexcr.2007.1002.1032.

4. Demars J, Cano M, Drouilhet L, Plisson-Petit, F, Bardou P, Fabre S, et al. Genome-Wide Identification of the Mutation Underlying Fleece Variation and Discriminating Ancestral Hairy Species from Modern Woolly Sheep. Mol Biol Evol. 2017; 34:1722-1729.

5. Erbe M, Hayes BJ, Matukumalli LK, Goswami S, Bowman PJ, Reich M, et al. Improving accuracy of genomic predictions within and between dairy cattle breeds with imputed high-density single nucleotide polymorphism panels. J Dairy Sci. 2012;95:4114–4129.

6. Purcell S, Neale B, Todd-Brown K, Thomas L, Ferreira MAR, Bender D, et al. PLINK: A Tool Set for Whole-Genome Association and Population-Based Linkage Analyses. Am J Hum Gene. 2007;81:559–575.

7. Gilmour AR, Gogel BJ, Cullis BR, Thompson R: ASReml User Guide Release 3.0. VSN Hemel Hempstead:International Ltd. 2009.

8. Duverger O. MMI. To grow or not to grow: Hair morphogenesis and human genetic hair disorders Semin. Cell Dev Bio. 2014;0:22–33.

9. Kayserili H. Uz E, Niessen C, Vargel I, Alanay Y, Tuncbilek G, et al. ALX4 dys-function disrupts craniofacial and epidermal development. Hum Mol Genet. 2009;18:4357–4366.

10. Kijas JW, Lenstra J, Hayes BJ, Boitard S, Porto Neto L, San Cristobal M, et al. Genome-wide analysis of the world's sheep breeds reveals high levels of historic mixture and strong recent selection. PLoS Biol. 2012;10:e1001258.

**Table 1 Genomic heritability and polygenic variance explained by variants in Published-Gene** **(V_Pub_) or Random-Gene sets of variants (V_Rand_) as a proportion of phenotypic (V_P_) and genomic variance (**V_(50k+Pub)_ or V_(50k+Rand)_) **for each trait using GBLUP.** The published (Pub) or random (Rand) sets were fitted as a separate GRM in addition to a GRM generated from 50K genotypes.

|  |  | hair/fleece genes | |  | random genes^a^ | |  |
| --- | --- | --- | --- | --- | --- | --- | --- |
| trait | h^2^ (V_50k+Pub_/V_P_) | V_Pub_/V_P_ | V_Pub_/V_(50k+Pub)_ |  | V_Rand_/V_P_ | V_Rand_/V_(50k+Rand)_ | ∆^b^ |
| ygfw | 0.38 (0.025) | 0.03 (0.015) | 0.07 (0.040) |  | 0.04 (0.003) | 0.11 (0.007) | -0.04 |
| agfw | 0.51 (0.030) | 0.04 (0.023) | 0.09 (0.044) |  | 0.03 (0.007) | 0.05 (0.013) | 0.04 |
| ycfw | 0.37 (0.026) | 0.04 (0.017) | 0.11 (0.045) |  | 0.04 (0.005) | 0.10 (0.014) | 0.01 |
| acfw | 0.48 (0.037) | 0.03 (0.030) | 0.07 (0.062) |  | 0.04 (0.009) | 0.08 (0.019) | -0.01 |
| ysl | 0.54 (0.030) | 0.08 (0.023) | 0.15 (0.042) |  | 0.05 (0.008) | 0.09 (0.015) | 0.06 |
| asl | 0.51 (0.036) | 0.10 (0.030) | 0.18 (0.058) |  | 0.04 (0.009) | 0.08 (0.017) | 0.10 |
| yfd | 0.65 (0.022) | 0.09 (0.019) | 0.15 (0.029) |  | 0.06 (0.006) | 0.08 (0.009) | 0.07 |
| afd | 0.69 (0.031) | 0.10 (0.031) | 0.15 (0.044) |  | 0.06 (0.014) | 0.09 (0.020) | 0.06 |
| ydcv | 0.44 (0.026) | 0.07 (0.018) | 0.16 (0.040) |  | 0.05 (0.008) | 0.12 (0.018) | 0.04 |
| adcv | 0.48 (0.039) | 0.10 (0.030) | 0.21 (0.063) |  | 0.06 (0.009) | 0.13 (0.018) | 0.08 |
| ycuv | 0.47 (0.031) | 0.08 (0.024) | 0.17 (0.050) |  | 0.07 (0.005) | 0.14 (0.010) | 0.03 |
| acuv | 0.60 (0.035) | 0.11 (0.030) | 0.18 (0.050) |  | 0.05 (0.013) | 0.08 (0.021) | 0.10 |
| yss | 0.26 (0.029) | 0.08 (0.021) | 0.30 (0.082) |  | 0.04 (0.008) | 0.16 (0.031) | 0.14 |
| ass | 0.32 (0.038) | 0.07 (0.028) | 0.23 (0.088) |  | 0.02 (0.007) | 0.06 (0.021) | 0.17 |
| ebwr | 0.37 (0.023) | 0.05 (0.016) | 0.13 (0.041) |  | 0.04 (00.007) | 0.12 (0.018) | 0.01 |
| ebcov | 0.11 (0.020) | 0.00 (0.000) | 0.0 (0.000) |  | 0.01 (0.003) | 0.09 (0.029) | -0.09 |

^b^difference in genetic variance in Published-Gene and Random-Gene sets of variants

^a^There were ten Random-Gene sets of variants and the results were averaged in the table above

V_(50k+Pub)_ is variance explained by 50k SNPs and variants in Published-Gene set

V_(50k+Rand)_ is variance explained by 50k SNPs and variants in Random-Gene set.

**Table 2 Variants with strong BayesR posterior probability of affecting at least one wool trait (pp ≥ 0. 5^a^). The BayesR model included all Illumina Ovine 50K SNPs as well as up-stream, down-stream and missense sequence variants for genes previously reported to affect wool or hair growth. The *P* values for the same variant from the multi-trait GWAS (M-GWAS) is also provided.**

^a^ posterior probability ≥ 0.2 is shown int the Table to indicate possible pleiotropic patterns and the empty cells are posterior probabilities of (pp) < 0.2

^b^ upstream and downstream regions of the gene were defined as within 5kb of the start and end position of the gene.
